# Bioluminescence Assays for Monitoring Chondrogenic Differentiation and Cartilage Regeneration

**DOI:** 10.3390/s17061306

**Published:** 2017-06-06

**Authors:** Hyeon Jeong Je, Min Gu Kim, Hyuck Joon Kwon

**Affiliations:** Department of Physical Therapy and Rehabilitation Science, College of Health Science, Eulji University, Gyeonggi 13135, Korea; runbee38@hanmail.net (H.J.J.); kevinzoq@naver.com (M.G.K.)

**Keywords:** bioluminescence assays, cartilage regeneration, chondrogenesis, real-time monitoring, in vivo bioluminescence imaging

## Abstract

Since articular cartilage has a limited regeneration potential, for developing biological therapies for cartilage regeneration it is important to study the mechanisms underlying chondrogenesis of stem cells. Bioluminescence assays can visualize a wide range of biological phenomena such as gene expression, signaling, metabolism, development, cellular movements, and molecular interactions by using visible light and thus contribute substantially to elucidation of their biological functions. This article gives a concise review to introduce basic principles of bioluminescence assays and applications of the technology to visualize the processes of chondrogenesis and cartilage regeneration. Applications of bioluminescence assays have been highlighted in the methods of real-time monitoring of gene expression and intracellular levels of biomolecules and noninvasive cell tracking within animal models. This review suggests that bioluminescence assays can be applied towards a visual understanding of chondrogenesis and cartilage regeneration.

## 1. Introduction

Articular cartilage is a type of vascular connective tissue and thus cannot be accessed by blood supply and mesenchymal stem cells (MSCs) originating from the bone marrow. Hence, cartilage tissues have limited regeneration potential [1,2]. Regenerative medicine and technology based on stem cells can be an attractive strategy to repair cartilage damage in patients. For developing biological therapies for cartilage regeneration, it is important to understand the mechanisms underlying chondrogenesis of stem cells [3].

Molecular imaging enables the visual monitoring, characterization and quantitative analysis of biological processes within living organisms [4]. Thus, molecular imaging can be a powerful research tool for studying mechanisms for chondrogenesis of stem cells and developing stem-cell therapy for cartilage regeneration. Among molecular imaging techniques, bioluminescence and fluorescence-based imaging techniques have been developed with the development of cameras with highly sensitive photosensors and have their applications in living organisms [5]. Fluorescence imaging requires external light absorption for light emission at a longer wavelength, whereas bioluminescence imaging requires the oxidation of luciferin catalyzed by luciferases to release photons of light [6,7]. Advanced molecular imaging systems including the development of a cooled charged coupled device (CCCD) camera allow us to quantify photons both in vitro and in vivo despite their light-absorbing and scattering properties [4]. Thus, the combination of optical systems and bioluminescence reporter systems enables us to monitor a variety of biological processes in real-time [8]. Real-time monitoring based on these advanced bioluminescence technologies can observe biological processes continuously, instead of at discrete time points, and thus provide the essential information for understanding tissue regeneration which is the sequential processes. Therefore, researchers are now trying to apply bioluminescence assays to regenerative medicine and its clinical applications [9]. This article will review the applications of bioluminescence assays to clarify underlying mechanisms of chondrogenesis of stem cell and stem cell-based therapy for cartilage regeneration. 

## 2. Fundamentals of Bioluminescence Assays

Fluorescence requires high-intensity excitation light which is inherently toxic for live cells and thus can lead to artifacts and abnormal responses. Thus, fluorescence imaging is not suitable for monitoring long-term processes due to continuous irradiation, causing cell damage and photobleaching (Table 1). In contrast, bioluminescence, which results from the activity of luciferase to catalyze oxidation of luciferins, has disadvantages such as lower brightness than fluorescence, requirement of luciferin and thus dependence on alterations to luciferin uptake rates due to changes in cell health, cellular location, and metabolic rates. However, it does not require excitation light, thereby having a low phototoxicity ([7], Table 1). Therefore, bioluminescence imaging is very useful for monitoring long-term processes of stem cell differentiation. Moreover, bioluminescence imaging offers higher sensitivity than fluorescent imaging due to the extremely low background signal ([6], Table 1). Until now, the *Photinus pyralis* (firefly) luciferase (Fluc), the *Renilla reniformis* luciferase (Rluc) and the *Gaussia princeps* luciferase (Gluc) have been widely used in biomedical research. Fluc converts D-luciferin to oxyluciferin by using ATP and Mg^2+^ as cofactors, resulting in green light emission at 562 nm [10], while Rluc uses coelenterazine to emit a blue light at 482 nm without any cofactors but has a significantly lower quantum yield and less enzymatic efficiency than Fluc [11]. Gaussia luciferase (Gluc) is a naturally secreted luciferase to emit the blue bioluminescence (480 nm peak) [12]. Although Gluc is much more sensitive than Fluc and Rluc, Gluc is less suitable for in vivo imaging because Gluc is highly absorbed by hemoglobin and melanin or is scattered by various tissues in living organisms [13]. The red-emitting luciferases from *Pyrophorus plagiophthalamus* [14], *Photinus pyralis* [15], *Luciola italica* [16] and railroad worm are known to be useful for the molecular imaging of deep tissues. Moreover, multicolor bioluminescence reporters based on beetle luciferases to emit green, orange, and red light allowed the simultaneous monitoring of the expression of multiple genes [17]. Recently, it was demonstrated that the bacterial bioluminescence *lux* system consisting of five genes (*luxCDABE*) can produce fully autonomous bioluminescence in mammalian cells without the addition of any chemical substrates [18]. The utility of these bioluminescence reporters allows for analysis of various aspects of biological functions, not only gene expression but also quantity of biomolecules, post-translational modification, and protein–protein interaction [19].

## 3. Real-Time Monitoring of Gene Expression

### 3.1. Principles

**Figure 1:**
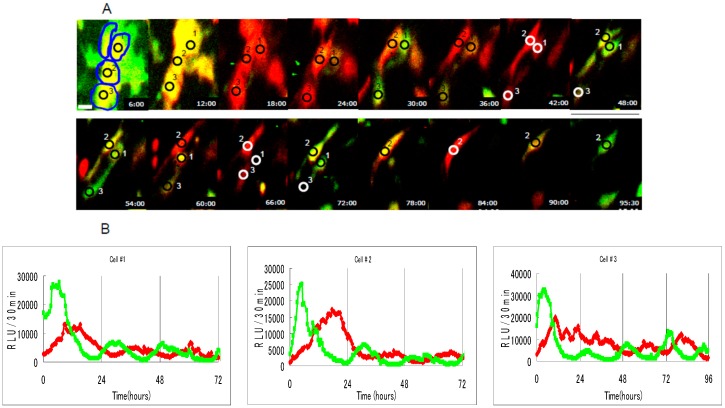
Bioluminescence imaging of dual gene expression at the single-cell level. (**A**) The dual-color bioluminescence imaging of NIH3T3 cells cotransfected with the Bmal1-red luciferase plasmid (red) and the Per2-green luciferase plasmid (green), showing circadian rhythms of green and red luminescence, respectively. Each spot is on a separate cell. Scale bar, 20 μm; (**B**) Representative circadian bioluminescence rhythms from individual NIH3T3 cells cotransfected with Bmal1-pSLR plasmid (red) and Per2-pEluc plasmid (green) for 3–4 days. Each graph represents real-time analysis data of quantitative bioluminescence intensity for the respective spot shown in Figure 1A. Reproduced with permission from [25].

Reporters consisting of the reporter genes under the control of a selected gene promoter have been used to analyze the transcriptional activities [20]. In comparison to typical reporter enzymes such as green fluorescent protein, β-galactosidase, and chloramphenicol acetyltransferase, luciferases have high sensitivity due to greatly reduced background, thereby providing the superior linear response range and thus are the most useful reporter enzymes for the quantitative analysis of gene expression [19]. Thus, bioluminescence reporter assays have been widely used in measuring gene expression levels in a variety of biological and pathological states from embryo development to disease progression. In general bioluminescence reporter assays, the luciferase reporters are transfected into target cells, the transfected cells expressing luciferases are lysed after an appropriate period, and then the quantitative levels of the expressed luciferase are measured by detecting a light signal to estimate the target promoter activity as a light intensity [21]. Thus, bioluminescence reporter assays have shortcomings such as requirement of cell lysis and time delays between luciferin application and bioluminescent signal acquisition. However, a molecular imaging system based on a CCCD camera and the bright bioluminescence reporters enables real-time monitoring of the level, the localization, and the duration of the gene expression without cell lysis, which is required for investigating dynamic processes of cell differentiation and tissue regeneration [22]. Furthermore, advanced luciferase technology, involving progress in both the multicolor reporter system and the detection equipment, has allowed us to simultaneously monitor the expressions of multiple genes because luciferases can emit different colored light in the catalysis of a single D-luciferin substrate and each of the intensities can be quantified by splitting these mixed emission spectra with optical filters (Figure 1) [23,24,25]. 

### 3.2. Application into Cartilage Researches

**Figure 2:**
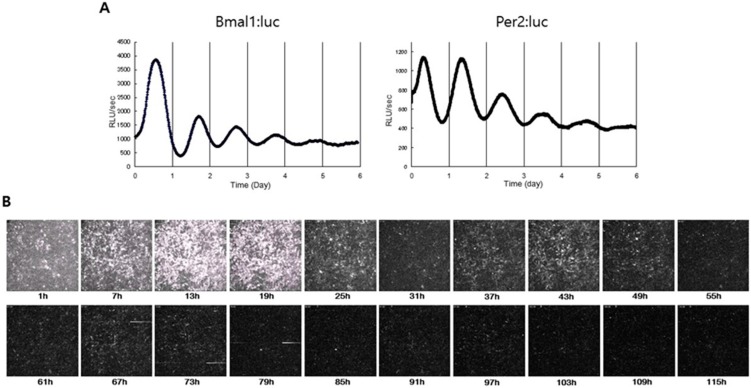
Bioluminescence imaging of circadian rhythms in chondrogenic cells. (**A**) The bioluminescence monitoring of ATDC5 cells transfected with the Bmal1-pEluc plasmid or the Per2-pEluc plasmid, shows circadian rhythms. (**B**) The single-cell imaging of ATDC5 cells transfected with the Bmal1-pEluc plasmid shows circadian rhythms in individual cells.

**Figure 3:**
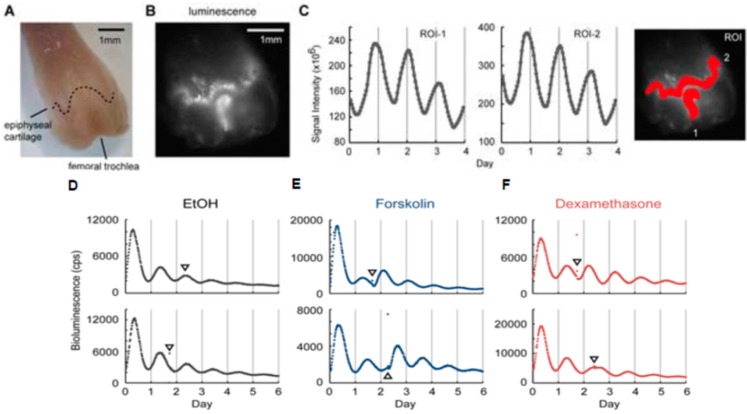
Bioluminescence imaging of circadian rhythms in Per2-Luc mouse femur. (**A**) The overview of the distal half of the femur. The dotted line indicates epiphyseal cartilage. The mirror-reversed image was used for comparison to the bioluminescence image. A sample was obtained from a 16.5-week old mouse and cultured for 6 days before observation. (**B**) The bioluminescence image obtained by a microscope-based high sensitivity CCCD camera system. (**C**) A time series analysis of the epiphyseal cartilage (**ROI-1**) and femoral trochlea (**ROI-2**). The right panel shows set ROIs. (**D–F**) Effects of forskolin and dexamethasone (DEX) on circadian clocks. Representative data showing the phase advancement (**upper panels**) or phase delay of the bone circadian clock (**lower panels**). The arrowhead indicates chemical administration. From left to right, the vehicle (ethanol; EtOH) (**D**), DEX (**E**), or forskolin (**F**). Reproduced with permission from [30].

The discovery of clock genes and their organization into transcription–translation feedback loops has motivated the development of techniques to monitor messenger ribonucleic acid (mRNA) and protein levels over time. Thus, bioluminescence reporters of clock genes which consist of the luciferase genes under the promoters of clock genes were incorporated into cell or animal models as indicators of clock function [24]. In mammalian research, firefly luciferase has been used typically under the control of promoter elements from the Period1 (Per1), Period2 (Per2) or brain and muscle arnt-like 1 (Bmal1) genes [24]. Dual-color bioluminescence reporter systems using beetle luciferases enabled us to monitor simultaneously Bmal1 and Per2, which are responsible for circadian rhythm [25]. Bioluminescence imaging reported the peripheral tissues exhibit robust circadian rhythms in culture by monitoring gene expression of clock genes [26]. More recently, bioluminescence imaging found autonomous circadian rhythms in explant cultures of xiphoid and femoral head cartilage and that temperature cycles were able to entrain the cartilage circadian rhythm [27], indicating that the temperature response could provide a mechanism by which the central clock can synchronize cartilage rhythms. Our study showed that chondrogenic cell line, ATDC5, which was stably transfected with either the Bmal1-Luc reporter or the Per2-Luc reporter, revealed robust circadian rhythms by dexamethasone treatment but the amplitude of circadian rhythms gradually decreased over time (Figure 2A). Subsequently, we performed single-cell imaging of the rhythmic expression of Bmal1 in the chondrogenic cells by using Brazilian click beetle luciferase (Eluc) which reveals a much brighter signal than Fluc [28]. The bioluminescence imaging of ATDC5 cells stably transfected with the Bmal1-Eluc reporter found that Bmal1 oscillations in individual cells were synchronized by dexamethasone treatment but showed the decrease in their amplitudes and the gradual desynchronization over time (Figure 2B). Furthermore, bioluminescence imaging of chondrogenic cells also showed that the circadian clock was strongly reset by parathyroid hormone in a circadian time-dependent manner [29]. In addition, the study using a Per2-Luc transgenic mouse showed that Per2 expression continues to oscillate with circadian rhythms in the articular cartilage tissues for several months (Figure 3A–C) and that cartilage clocks were reset by forskolin and dexamethasone in a time-specific manner (Figure 3D–F), which suggests that hormones such as glucocorticoids play a role as internal time-cues for the circadian clock in cartilage tissues [30].

Bioluminescence imaging, which can monitor changes in gene expression of cells implanted in animal models, facilitates the development of cell therapies for tissue regeneration. In cartilage development, type II procollagen (COL2A1) gene expression is known to be upregulated by sex determining region Y-box (Sox) 9, L-Sox5 and Sox6 in response to environmental signals [31], which suggests that COL2A1 is a marker of chondrogenesis [32]. To monitor cartilage formation in an animal model, progenitor cells were transfected with both the Fluc under the control of the COL2A1 promoter as a reporter of chondrogenic marker and Rluc under the control of a cytomegalovirus promoter as a reporter of cell proliferation [33]. In vivo bioluminescence monitoring also showed that the Pluc/Rluc ratio represents changes in gene expression of aggrecan in the mice implanted with the CL1 cell line and MSCs, which revealed different patterns of in vivo chondrogenesis. In addition, it was shown that both bioluminescent signal intensity and area decreased with natural aging from 2 to 13 months, indicating that the bioluminescence intensity can be used as a quantitative indicator of regenerated tissues during cartilage regeneration [34]. 

## 4. Realtime Monitoring of Biomolecules

### 4.1. Principles

ATP serves numerous vital functions as the central molecule of metabolism in the cell because molecules used as energy sources, for instance glucose, are broken down in the cell, and the energy obtained from them is stored in the phosphate-anhydride bonds of ATP [35]. ATP also maintains the proper concentrations of other nucleotides. ATP is interchanged into the other NTPs, which are incorporated into DNA and RNA. In addition, extracellular ATP functions as an autocrine/paracrine signaling molecule which regulates many physiological functions [36]. Ligand-gated ionic channels, P2X receptors, which have been identified mainly in neurons and muscle cells, respond to ATP directly by activating depolarizing currents [37,38]. Thus, ATP detection assays are crucial for understanding many physiological and pathological conditions [39,40,41]. Beetle luciferases which catalyze the oxidation of the luciferin by using ATP in the presence of O^2^ and Mg^2+^ to emit luminescence have been widely used as an excellent ATP reporter for detecting intracellular ATP levels [6,7]. Previous studies showed that the beetle luciferases under the control of a constitutive promoter can be used for monitoring intracellular ATP level in real time [42,43], which indicates that the beetle luciferases can continuously monitor intracellular ATP levels. Luciferase-based assays can also detect ATP-converting reactions such as oxidative phosphorylation, photophosphorylation [44]. 

Ca^2+^ is an intracellular signal molecule that regulates differentiation, secretion, contraction, cellular excitability and gene expression. Ca^2+^ regulates many intracellular processes in the cytosol and inside organelles such as endoplasmic and sarcoplasmic reticulums, mitochondrias, endosomes, Golgi apparatus, and lysosomes [45,46]. Therefore, accurate measurement of the Ca^2+^ levels inside the organelles is important for understanding the physiological functions of Ca^2+^ signals. Since aequorin, which is the protein-based Ca^2+^ indicator isolated from the jellyfish *Aequorea forskalea*, was firstly used in the early 1970s [47], several Ca^2+^-binding photoproteins have been used to measure Ca^2+^ levels [48,49]. In comparison to synthetic fluorescence dyes, the advantage of the protein-based Ca^2+^ indicators is their ability to be targeted to specific intracellular locations by coupling to specific promoters and targeting sequences [50]. Furthermore, the protein-based Ca^2+^ indicators enable not only long-term imaging of Ca^2+^ signals in specific subcellular compartments and intact living animals, but also repeated imaging of the same living organisms. Aequorin emits blue light (465 nm) when Ca^2+^ binds to at least two sites among three Ca^2+^-binding sites of aequorin [51], the molecular oxygen in aequorin is released, and then the coelenterazine is oxidized to coelenteramide [52]. Thus, aequorin has been widely used for detecting Ca^2+^ levels in living organisms [53,54,55]. Recent work also developed fluorescent Ca^2+^ sensors by combining an improved GFP variant and aequorin [56]. 

Cyclic adenosine 3′,5′-monophosphate (cAMP) functions as one of the principal signal molecules by regulating a number of signal pathways, including those activated by G protein-coupled receptors in response to hormones and neurotransmitters [57]. Thus, biochemical cAMP assays have been developed for basic research and drug discovery. The cAMP reporter based on bioluminescent enzymes used N-terminal fragments of a click beetle luciferase from Brazil (ELucN) and one from Jamaica (CBRN) and one C-terminal fragment of carboxy-terminal fragment engineered from click beetle luciferase (McLuc1) which dimerize with the above *N*-terminal fragments, forming two distinct luminescent enzymes with different emission peaks well-separated from each other [58]. In this cAMP reporter, the cAMP-binding domain of PKA (RIIβ) was used for sensing cAMP levels. McLuc1 and ElucN form a functional enzyme to emit red light (613 nm) in the absence of cAMP. cAMP binding to PKA RIIβ of the cAMP reporter results in conformational rearrangement, driving separation of the red light-producing luciferase and migration of McLuc1 toward CBRN, which consequently emits green light (538 nm). Thus, cAMP levels could be expressed by red/green ratio. Other cAMP luminescent indicators were also developed by using a circularly permutated variant of a Fluc fused with cAMP-binding domain B of PKA RIIβB [59,60]. These cAMP reporters are very highly-sensitive cAMP sensors with a detection limit in low nanomolar range and also have high signal/noise ratio [60], which enables real-time monitoring of cAMP dynamics in living organisms.

### 4.2. Application to Cartilage Research

**Figure 4:**
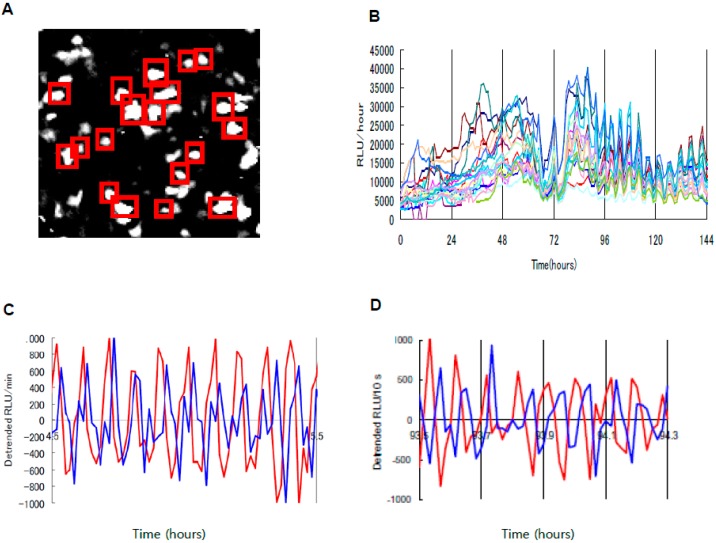
Bioluminescence imaging of ATP, Ca^2+^ and oxygen during chondrogenesis. (**A**) Right panel: Bioluminescence imaging at the single-cell level shows that bioluminescent intensities of Actin-*Phrixothrix hirtus* red luciferase (PxRe) to report intracellular ATP levels in individual cells oscillate collectively by intercellular synchronization during chondrogenesis. Left panel: Time course data of P_ACTIN_-PxRe intensities of individual cells indicated by red squares after chondrogenic induction. Reproduced with permission from [65]. (**B**) Simultaneous monitoring of intracellular oxygen (blue line) and ATP (red line) levels during chondrogenesis in the micromass culture of mesenchymal stem cells (MSCs) by using an oxygen reporter (P_ACTIN_-Rluc) and ATP reporter (P_ACTIN_-PxRe). Reproduced with permission from [65]. (**C**) Simultaneous monitoring of intracellular Ca^2+^ (blue line) and ATP (red line) levels during chondrogenesis in micromass culture of ATDC5 cells using a calcium reporter (aequorin) and ATP reporter (P_ACTIN_-PxRe). Reproduced with permission from [67].

Continuous monitoring of biomolecules during chondrogenesis offers the exciting possibility to clarify the mechanism underlying cartilage regeneration. Prechondrogenic condensation is the most critical process for skeletal patterning during limb development [61,62]. Since the secreted molecules such as adhesion molecules and extracellular matrix (ECM) are considered to be strictly controlled to determine the patterns of condensations during cartilage formation, how secretory activity is regulated during chondrogenesis was examined by monitoring the dynamics of intracellular ATP which is required for secretion processes [63,64]. Bioluminescence monitoring using a *Phrixothrix hirtus* red luciferase (PxRe)-based ATP reporter showed that intracellular ATP level oscillates in chondrogenesis ([65]; Figure 4A). In addition, a dual-color bioluminescence assay which simultaneously monitors both ATP and oxygen levels in real time was developed by using PxRe to emit red and Rluc to emit blue light [66]. In this dual-color monitoring analysis, Rluc oscillations revealed troughs roughly corresponding with the peak of PxRe oscillations during chondrogenesis (Figure 4B), which can be explained by the fact that ATP is synthesized by oxygen consumption in mitochondria. However, Rluc oscillations also had an additional mode with a large phase difference relative to PxRe oscillations (Figure 4B), indicating the oscillations of non-mitochondrial oxygen consumption in chondrogenesis [67]. Since it was demonstrated that metabolic intermediates involved in glycolysis oscillate during chondrogenesis [65], the glycolytic oscillations may drive the non-mitochondrial oxygen oscillations. However, oxygen consumption by peroxidases and plasma membrane-bound NADPH-oxidase may be involved in non-mitochondrial oxygen oscillations.

Similarly, by using a blue-emitting aequorin and a red-emitting PxRe luciferase, intracellular Ca^2+^ and ATP levels were simultaneously monitored during chondrogenesis [67]. The results showed that both Ca^2+^ and ATP levels oscillated and Ca^2+^ oscillations were nearly antiphase to ATP oscillations ([67]; Figure 4C). It was known that Ca^2+^ stimulates Ca^2+^ pumps and other intracellular reactions such as exocytosis, which leads to ATP consumption. Moreover, Ca^2+^ can suppress ATP synthesis by inhibiting glycolytic enzymes and collapsing mitochondrial membrane potential [68,69]. Likewise, the decrease in Ca^2+^ level can reduce ATP consumption and increase ATP production [70]. This positive Ca^2+^ effect on ATP consumption and the negative Ca^2+^ effect on ATP production can explain the antiphase relationship between Ca^2+^ and ATP oscillations.

## 5. Real-Time Monitoring of Exocytotic Activity

### 5.1. Principles

Molecular imaging of exocytotic activities in cells has used total internal reflection fluorescence and two-photon laser scanning microscopy [71,72]. However, these fluorescence methods have limitations in monitoring a limited section of the cells and require continuous light excitation to cause cellular toxicity and photobleaching. Bioluminescence imaging can provide distinct advantages over fluorescence imaging in monitoring protein secretion and other secretory processes in cells because bioluminescence imaging does not require light excitation and thus has no phototoxicity. The visualization of secretion of *Cypridina* luciferase (Cluc) and *Gaussia* luciferase (GLuc) was realized in mammalian cells, showing that the secreted luciferases are secreted via the constitutive exocytotic pathways [73,74]. Cluc was thus utilized for imaging neurotransmitter release [75].

### 5.2. Application to Cartilage Research

**Figure 5:**
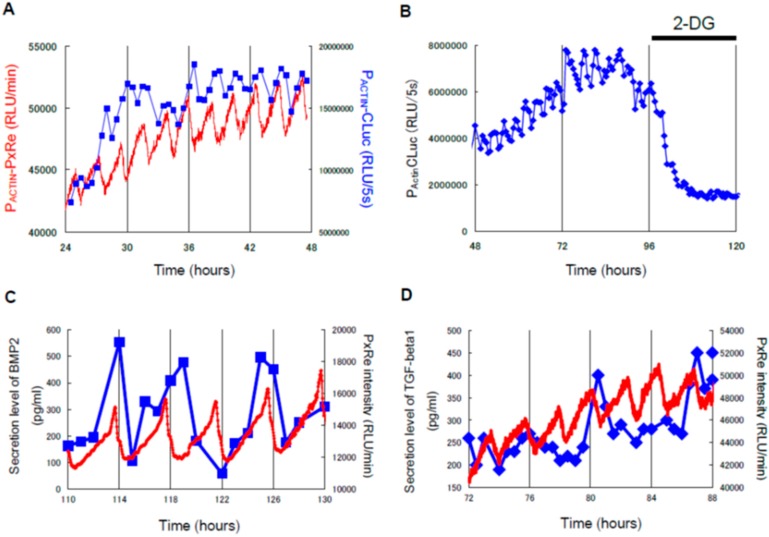
Simultaneous monitoring of secretory activity and intracellular ATP levels during chondrogenesis. (**A**) Time course data taken with simultaneous monitoring of PxRe intensity (red line) and secreted *Cypridina* luciferase (CLuc) intensity (blue line) during perfusion with insulin-implemented medium. Reproduced with permission from [65]. (**B**) Effect of 2-deoxy glucose (2-DG) treatment on the oscillatory secretion of CLuc during perfusion with insulin-implemented medium. Reproduced with permission from [65]. (**C**) Simultaneous monitoring of PxRe intensity (red line) and secreted BMP2 levels (blue line) during perfusion with the chondrogenic medium. Reproduced with permission from [76]. (**D**) Simultaneous monitoring of PxRe intensity (red line) and secreted TGF-β1 levels (blue line) during perfusion with chondrogenic medium. Reproduced with permission from [67]. Reproduced with permission from [76].

Since the secreted molecules including ECM and adhesion molecules must be strictly controlled to determine the skeletal patterns during chondrogenesis, it was examined how secretion activity is regulated during chondrogenesis. Secretory activity was monitored by using a reporter based on the Cluc gene fused to a constitutive promoter. The bioluminescence monitoring system combined with the perfusion culture system was used for simultaneous monitoring of both intracellular ATP level and secreted Cluc levels during chondrogenesis. The result revealed that Cluc secretion oscillated during chondrogenesis ([65]; Figure 5A). The oscillation period of Cluc secretion was the nearly same period ATP oscillations. In addition, 2-deoxy glucose which eliminated ATP oscillations suppressed the Cluc oscillations ([65]; Figure 5B), indicating that the oscillatory secretion is driven by ATP oscillations in chondrogenesis. Furthermore, it was shown that secretion levels of Bone morphogenetic protein 2 (BMP2) and transforming growth factor-β1 (TGF-β1) oscillated during chondrogenesis and that each peak of the oscillatory secretion of BMP2 and TGF-β1 appeared at the peak of ATP oscillations ([76]; Figure 5C,D). However, the oscillatory secretion of the growth factors showed one peak per two or three peaks of ATP oscillations and thus the frequency of their oscillatory secretion was lower than that of ATP oscillations ([76]; Figure 4C,D). This result indicates that the secretion patterns of the growth factors depend on not only secretory activity but also other processes. The oscillatory secretion of growth factors would play a crucial role in prechondrogenic condensation and subsequent skeletal patterning.

## 6. In Vivo Imaging for Transplanted Cells

### 6.1. Principles

Cell therapies hold great promise for tissue regeneration. However, before clinical application of the cell therapies, transplanted cells must be monitored in vivo for understanding the mechanism underlying tissue regeneration. In vivo bioluminescence imaging enables quantitative and repetitive measurements of transplanted cells in animal models without a light source to excite fluorophores and thus provides useful information on cell survival, migration, and proliferation and differentiation over time in the same animal models [77]. For example, after transplantation into a murine myocardial infarction model, bone marrow mononuclear cells, mesenchymal stem cells, adipose stromal cells, and skeletal myoblasts which were labelled with bioluminescence reporters were monitored in vivo. It was demonstrated that mononuclear cells revealed higher survival rate and induced better heart function than other cell types [78,79]. However, bioluminenscence imaging has limitaions in its application to in vivo studies due to the differences in bioluminescent output kinetics among subcutaneous injection, tail vein injection and intraperitoneal injection, and the stress and wounding effects associated with repeated luciferin injection. To overcome this limitation, bioluminescence techniques based on the bacterial *lux* system were developed to produce fully autonomous bioluminescence in a human cell line without the injection of any exogenous substrate [18]. 

### 6.2. Application to Cartilage Research

**Figure 6:**
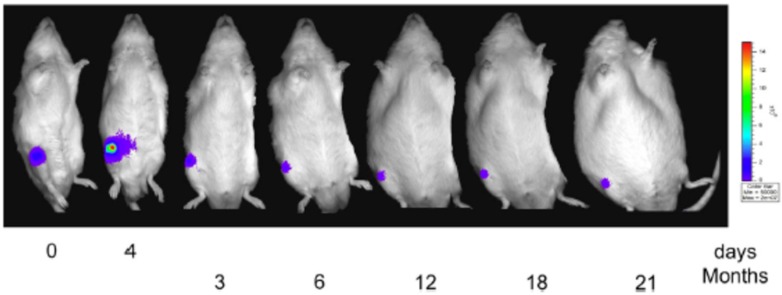
CCD images from the long-term observation of luciferase activity from the right knee joint. This representative image shows the CCD image of one rat from the AC-SY group (transplanted with chondrocyte and synovial cell sheets) at the following time points (from left to right): after transplantation on day 0 and day 4, and at 3, 6, 12, 18, and 21 months. The color bar indicates the bioluminescence intensity in photons per seconds cm^2^ per steradian. Reproduced with permission from [80].

Bioluminescence imaging was used for in vivo cell tracking after the cell sheets made of firefly luciferase-expressing chondrocytes obtained from transgenic rats were transplanted into the knee joint of rats for cartilage regeneration [80]. Bioluminescence imaging showed that the transplanted cells remained in the knee joint and did not migrate to other parts of the body, which confirms the safety of the chondrocyte sheets ([80]; Figure 6). In vivo bioluminescence imaging also examined the potential effect of gene delivery on cartilage treatment. The in vivo imaging showed that the adeno-associated virus-mediated intra-articular transgene can be stably expressed through a single intra-articular injection and can be regulated by using a tetracycline-inducible system in a rat model [81], which indicates that the adeno-associated virus-mediated system has a clinical potential for inflammatory and degenerative arthritis. 

## 7. Potential Applications of Bioluminescence Assays for Cartilage Regeneration

Understanding how proteins interact during cartilage formation is crucial for biological and medical research on cartilage regeneration. Bioluminescence resonance energy transfer (BRET) which is based on energy transfer between a donor and an acceptor is useful for protein–protein interaction [82]. BRET allows the detection of interactions between fusion proteins without external fluorescence excitation [83], and thus could identify protein–protein interaction, which play crucial roles for chondrogenesis. In addition, bioluminescence imaging can monitor successfully the post-transcriptional events such as RNA processing and splicing, which are known to regulate cartilage formation. For example, mRNA stability can be monitored by fusing a luciferase reporter to the 3’ untranslated region of an interested gene [84]. Furthermore, the continuous improvement of the *lux* system to produce a bioluminescent signal without exogenous substrate for functions in eukaryotic cells will be useful in basic and applied scientific research for cartilage regeneration. Further study using these bioluminescence systems will provide the useful information for understanding the mechanism underlying chondrogenic differentiation and cartilage regeneration. 

## 8. Conclusions

Bioluminescence assays can be applied readily into most cell and tissue types because luciferases can be expressed and luciferin easily permeates into most cells and tissue types, which makes it a versatile technology for a variety of biomedical research [8]. Furthermore, in vivo bioluminescence imaging has become a powerful technique for the noninvasive monitoring of animal models, which will provide insights into molecular mechanism underlying chondrogenic differentiation to cartilage regeneration. New applications of bioluminescence will continue to deepen our understanding of cartilage regeneration and consequently lead to therapeutic developments in the future.

## Figures and Tables

**Table 1:** Comparison of bioluminescence and fluorescence.

	Advantage	Disadvantage
Fluorescence	Superior brightness	Requirement of excitation light
	Shorter imaging time (miliseconds)	High phototoxicity
		High photobleaching
		High background
		Autofluorescence
		Autofluorescence
Bioluminescence	Low phototoxicity	Low brightness
	Low background	Longer imaging time (minute)
	High sensitivity	Requirement of substrates
	Broad linear range	

## Abbreviations

OA	osteoarthritis
CCCD	cooled charged coupled device
Fluc	*Photinus pyralis* (firefly) luciferase
Rluc	the sea pansy *Renilla reniformis* luciferase
Gluc	the marine copepod *Gaussia princeps* luciferase
PxRe	*Phrixothrix hirtus* red luciferase
Cluc	*Cypridina* luciferase
*lux*	the bacterial luciferase gene cassette
AAV	adeno-associated virus
FRET	fluorescence resonance energy transfer
BRET	bioluminescence resonance energy transfer

## References

[1] O’Driscoll S.W. (1998). Current concepts review—The healing and regeneration of articular cartilage. JBJS.

[2] Mandelbaum B.R., Browne J.E., Fu F., Micheli L., Mosely J.B., Erggelet C., Minas T., Peterson L. (1998). Articular cartilage lesions of the knee. Am. J. Sports Med..

[3] Zhang J., Dong S., Sivak W., Sun H.B., Tao K. (2017). Stem cells in cartilage regeneration. Stem Cells Int..

[4] Massoud T.F., Gambhir S.S. (2003). Molecular imaging in living subjects: Seeing fundamental biological processes in a new light. Genes Dev..

[5] Stephens D.J., Allan V.J. (2003). Light microscopy techniques for live cell imaging. Science.

[6] Badr C.E., Tannous B.A. (2011). Bioluminescence imaging: Progress and applications. Trends Biotechnol..

[7] Marques S.M., Esteves da Silva J.C. (2009). Firefly bioluminescence: A mechanistic approach of luciferase catalyzed reactions. IUBMB Life.

[8] Greer L.F., Szalay A.A. (2002). Imaging of light emission from the expression of luciferases in living cells and organisms: A review. Luminescence.

[9] Prescher J.A., Contag C.H. (2010). Guided by the light: Visualizing biomolecular processes in living animals with bioluminescence. Curr. Opin. Chem. Biol..

[10] LI J., Chen L., Du L., Li M. (2013). Cage the firefly luciferin!—A strategy for developing bioluminescent probes. Chem. Soc. Rev..

[11] Shimomura O., Teranishi K. (2000). Light-emitters involved in the luminescence of coelenterazine. Luminescence.

[12] Badr C.E., Hewett J.W., Breakefield X.O., Tannous B.A. (2007). A highly sensitive assay for monitoring the secretory pathway and ER stress. PLoS ONE.

[13] Wurdinger T., Badr C., Pike L., De Kleine R., Weissleder R., Breakefield X.O., Tannous B.A. (2008). A secreted luciferase for ex vivo monitoring of in vivo processes. Nat. Methods.

[14] Villalobos V., Naik S., Bruinsma M., Dothager R.S., Pan M.H., Samrakandi M., Moss B., Elhammali A., Piwnica-Worms D. (2010). Dual-color click beetle luciferase heteroprotein fragment complementation assays. Chem. Biol..

[15] Caysa H., Jacob R., Müther N., Branchini B., Messerle M., Söling A. (2009). A redshifted codon-optimized firefly luciferase is a sensitive reporter for bioluminescence imaging. Photochem. Photobiol. Sci..

[16] Branchini B.R., Southworth T.L., DeAngelis J.P., Roda A., Michelini E. (2006). Luciferase from the Italian firefly Luciola italica: Molecular cloning and expression. Comp. Biochem. Physiol. Biochem. Mol. Biol..

[17] Nakajima Y., Kimura T., Sugata K., Enomoto T., Asakawa A., Kubota H., Ikeda M., Ohmiya Y. (2005). Multicolor luciferase assay system: One-step monitoring of multiple gene expressions with a single substrate. Biotechniques.

[18] Close D.M., Patterson S.S., Ripp S., Baek S.J., Sanseverino J., Sayler G.S. (2010). Autonomous bioluminescent expression of the bacterial luciferase gene cassette (lux) in a mammalian cell line. PLoS ONE.

[19] Nakajima Y., Ohmiya Y. (2010). Bioluminescence assays: Multicolor luciferase assay, secreted luciferase assay and imaging luciferase assay. Expert Opin. Drug Discov..

[20] Robertson J.B., Johnson C.H. (2011). Luminescence as a continuous real-time reporter of promoter activity in yeast undergoing respiratory oscillations or cell division rhythms. Yeast Genet. Netw. Methods Protoc..

[21] Bronstein I., Fortin J., Stanley P.E., Stewart G.S., Kricka L.J. (1994). Chemiluminescent and bioluminescent reporter gene assays. Anal. Biochem..

[22] Hoshino H., Nakajima Y., Ohmiya Y. (2007). Luciferase-YFP fusion tag with enhanced emission for single-cell luminescence imaging. Nat. Methods.

[23] Yasunaga M., Nakajima Y., Ohmiya Y. (2014). Dual-color bioluminescence imaging assay using green- and red-emitting beetle luciferases at subcellular resolution. Anal. Bioanal. Chem..

[24] Welsh D.K, Kay S.A. (2005). Bioluminescence imaging in living organisms. Curr. Opin. Biotechnol..

[25] Kwon H., Enomoto T., Shimogawara M., Yasuda K., Nakajima Y., Ohmiya Y. (2010). Bioluminescence imaging of dual gene expression at the single-cell level. BioTechniques.

[26] Yoo SH., Yamazaki S., Lowrey P.L., Shimomura K., Ko C.H., Buhr E.D., Siepka S.M., Hong H.K., Oh W.J., Yoo O.J. (2004). PERIOD2: LUCIFERASE real-time reporting of circadian dynamics reveals persistent circadian oscillations in mouse peripheral tissues. Proc. Natl. Acad. Sci. USA.

[27] Gossan N., Zeef L., Hensman J., Hughes A., Bateman J.F., Rowley L., Little C.B., Piggins H.D., Rattray M., Boot-Handford R.P. (2013). The circadian clock in murine chondrocytes regulates genes controlling key aspects of cartilage homeostasis. Arthritis Rheum..

[28] Nakajima Y., Yamazaki T., Nishii S., Noguchi T., Hoshino H., Niwa K., Viviani V.R., Ohmiya Y. (2010). Enhanced beetle luciferase for high-resolution bioluminescence imaging. PLoS ONE.

[29] Hosokawa T., Tsuchiya Y., Okubo N., Kunimoto T., Minami Y., Fujiwara H., Umemura Y., Koike N., Kubo T, Yagita K. (2015). Robust Circadian Rhythm and Parathyroid Hormone-Induced Resetting during Hypertrophic Differentiation in ATDC5 Chondroprogenitor Cells. Acta Histochem. Cytochem..

[30] Okubo N., Minami Y., Fujiwara H., Umemura Y., Tsuchiya Y., Shirai T., Oda R., Inokawa H., Kubo T., Yagita K. (2013). Prolonged bioluminescence monitoring in mouse ex vivo bone culture revealed persistent circadian rhythms in articular cartilages and growth plates. PLoS ONE.

[31] Okazaki K., Sandell L.J. (2004). Extracellular matrix gene regulation. Clin. Orthop. Relat. Res..

[32] Lefebvre V., Huang W., Harley V.R., Goodfellow P.N., De Crombrugghe B. (1997). SOX9 is a potent activator of the chondrocyte-specific enhancer of the pro alpha1 (II) collagen gene. Mol. Cell. Biol..

[33] Vilalta M., Jorgensen C., Dégano I.R., Chernajovsky Y., Gould D., Noël D., Andrades J.A., Becerra J., Rubio N., Blanco J. (2009). Dual luciferase labelling for non-invasive bioluminescence imaging of mesenchymal stromal cell chondrogenic differentiation in demineralized bone matrix scaffolds. Biomaterials.

[34] Mailhiot S.E., Zignego D.L., Prigge J.R., Wardwell E.R., Schmidt E.E., June R.K. (2015). Non-invasive quantification of cartilage using a novel in vivo bioluminescent reporter mouse. PLoS ONE.

[35] Bridger W.A., Henderson J.F. (1983). Cell ATP.

[36] Gordon J.L. (1986). Extracellular ATP: Effects, sources and fate. Biochem. J..

[37] Del Puerto A., Wandosell F.G., Garrido J.J. (2013). Neuronal and glial purinergic receptors functions in neuron development and brain disease. Front. Cell. Neurosci..

[38] Coddou C., Yan Z., Obsil T., Huidobro-Toro J.P., Stojilkovic S.S. (2011). Activation and regulation of purinergic P2X receptor channels. Pharmacol. Rev..

[39] Lundin A. (2000). Use of firefly luciferase in ATP-related assays of biomass, enzymes, and metabolites. Methods Enzymol..

[40] Crouch S.P.M., Kozlowski R., Slater K.J., Fletcher J. (1993). The use of ATP bioluminescence as a measure of cell proliferation and cytotoxicity. J. Immunol. Methods.

[41] Dexter S.J., Cámara M., Davies M., Shakesheff K.M. (2003). Development of a bioluminescent ATP assay to quantify mammalian and bacterial cell number from a mixed population. Biomaterials.

[42] Ainscow E.K., Rutter G.A. (2002). Glucose-stimulated oscillations in free cytosolic ATP concentration imaged in single islet β-cells. Diabetes.

[43] Koop A., Cobbold P.H. (1993). Continuous bioluminescent monitoring of cytoplasmic ATP in single isolated rat hepatocytes during metabolic poisoning. Biochem. J..

[44] Turner D.E., Daugherity E.K., Altier C., Maurer K.J. (2010). Efficacy and limitations of an ATP-based monitoring system. J. Am. Assoc. Lab. Anim. Sci..

[45] Clapham D.E. (2007). Calcium signaling. Cell.

[46] Rizzuto R., Pozzan T. (2006). Microdomains of intracellular Ca2+: Molecular determinants and functional consequences. Physiol. Rev..

[47] Llinás R., Blinks JR., Nicholson C. (1972). Calcium transient in presynaptic terminal of squid giant synapse: Detection with aequorin. Science.

[48] Shimomura O. (1985). Bioluminescence in the sea: Photoprotein systems. Symp. Soc. Exp. Biol..

[49] Tsuji F.I., Ohmiya Y., Fagan T.F., Toh H., Inouye S. (1995). Molecular evolution of the Ca^2+^ binding photoproteins of the hydrozoa. Photochem. Photobiol..

[50] Hofer A.M., Machen T.E. (1993). Technique for in situ measurement of calcium in intracellular inositol 1,4,5-trisphosphate-sensitive stores using the fluorescent indicator mag-fura-2. Proc. Natl. Acad. Sci. USA.

[51] Inouye S., Aoyama S., Miyata T., Tsuji F.I., Sakaki Y. (1989). Overexpression and purification of the recombinant Ca2+-binding protein, apoaequorin. J. Biochem..

[52] Allen D.G., Prendergast F.G. (1977). Aequorin luminescence: Relation of light emission to calcium concentration—A calcium-independent component. Science.

[53] Rizzuto R., Simpson A.W., Brini M., Pozzan T. (1992). Rapid changes of mitochondrial Ca^2+^ revealed by specifically targeted recombinant aequorin. Nature.

[54] Montero M., Brini M., Marsault R., Alvarez J., Sitia R., Pozzan T., Rizzuto R. (1995). Monitoring dynamic changes in free Ca^2+^ concentration in the endoplasmic reticulum of intact cells. EMBO J..

[55] Brini M., Murgia M., Pasti L., Picard D., Pozzan T., Rizzuto R. (1993). Nuclear Ca^2+^ concentration measured with specifically targeted recombinant aequorin. EMBO J..

[56] Rodriguez-Garcia A., Rojo-Ruiz J., Navas-Navarro P., Aulestia F.J., Gallego-Sandin S., Garcia-Sancho J., Alonso M.T. (2014). GAP, an aequorin-based fluorescent indicator for imaging Ca^2+^ in organelles. Proc. Natl. Acad. Sci. USA.

[57] McKnight G.S. (1991). Cyclic AMP second messenger systems. Curr. Opin. Cell Biol..

[58] Takeuchi M., Nagaoka Y., Yamada T., Takakura H., Ozawa T. (2010). Ratiometric bioluminescence indicators for monitoring cyclic adenosine 3′,5′-monophosphate in live cells based on luciferase-fragment complementation. Anal. Chem..

[59] Fan F., Binkowski B.F., Butler B.L., Stecha P.F., Lewis M.K., Wood K.V. (2008). Novel genetically encoded biosensors using firefly luciferase. ACS Chem. Biol..

[60] Binkowski B.F., Butler B.L., Stecha P.F., Eggers C.T., Otto P., Zimmerman K., Vidugiris G., Wood M.G., Encell L.P., Fan F. (2011). A luminescent biosensor with increased dynamic range for intracellular cAMP. ACS Chem. Biol..

[61] Olsen B.R., Reginato A.M., Wang W. (2000). Bone development. Annu. Rev. Cell Dev. Biol..

[62] Mariani F.V., Martin G.R. (2003). Deciphering skeletal patterning: Clues from the limb. Nature.

[63] Martin T.F. (1997). Stages of regulated exocytosis. Trends Cell Biol..

[64] Jones P.M., Persaud S.J. (1998). Protein Kinases, protein Phosphorylation, and the regulation of insulin secretion from pancreatic β-Cells. Endocr. Rev.

[65] Kwon H.J., Ohmiya Y., Honma K.I., Honma S., Nagai T., Saito K., Yasuda K. (2012). Synchronized ATP oscillations have a critical role in prechondrogenic condensation during chondrogenesis. Cell Death Dis..

[66] Kwon H.J., Ohmiya Y., Yasuda K. (2014). Simultaneous monitoring of intracellular ATP and oxygen levels in chondrogenic differentiation using a dual-color bioluminescence reporter. Luminescence.

[67] Herst P.M., Tan A.S., Scarlett D.J.G., Berridge M.V. (2004). Cell surface oxygen consumption by mitochondrial gene knockout cells. BBA Bioenerg..

[68] Kwon H.J., Ohmiya Y., Yasuda K. (2012). Dual-color system for simultaneously monitoring intracellular Ca^2+^ and ATP dynamics. Anal. Biochem..

[69] Jung S.K., Kauri L.M., Qian W.J., Kennedy R.T. (2000). Correlated oscillations in glucose consumption, oxygen consumption, and intracellular free Ca^2+^ in single islets of Langerhans. J. Biol. Chem..

[70] Magnus G., Keizer J. (1998). Model of β-cell mitochondrial calcium handling and electrical activity. II. Mitochondrial variables. Am. J. Physiol. Cell Physiol..

[71] Detimary P., Gilon P., Henquin J.C. (1998). Interplay between cytoplasmic Ca^2+^ and the ATP/ADP ratio: A feedback control mechanism in mouse pancreatic islets. Biochem. J..

[72] Burchfield J.G., Lopez J.A., Mele K., Vallotton P., Hughes W.E. (2010). Exocytotic vesicle behaviour assessed by total internal reflection fluorescence microscopy. Traffic.

[73] Takahashi N., Kasai H. (2007). Exocytic process analyzed with two-photon excitation imaging in endocrine pancreas. Endocr. J..

[74] Inouye S., Ohmiya Y., Toya Y., Tsuji F.I. (1992). Imaging of luciferase secretion from transformed Chinese hamster ovary cells. Proc. Natl. Acad. Sci. USA.

[75] Suzuki T., Usuda S., Ichinose H., Inouye S. (2007). Real-time bioluminescence imaging of a protein secretory pathway in living mammalian cells using Gaussia luciferase. FEBS Lett..

[76] Miesenböck G., Rothman J.E. (1997). Patterns of synaptic activity in neural networks recorded by light emission from synaptolucins. Proc. Natl. Acad. Sci. USA.

[77] Kwon H.J., Han Y. (2015). Dual monitoring of secretion and ATP levels during chondrogenesis using perfusion culture-combined bioluminescence monitoring system. BioMed Res. Int..

[78] Cao Y.A., Wagers A.J., Beilhack A., Dusich J., Bachmann M.H., Negrin R.S., Weissman I.L., Contag C.H. (2004). Shifting foci of hematopoiesis during reconstitution from single stem cells. Proc. Natl. Acad. Sci. USA.

[79] Van der Bogt K.E., Sheikh A.Y., Schrepfer S., Hoyt G., Cao F., Ransohoff K.J., Swijnenburg R.J., Pearl J., Lee A., Fischbein M. (2008). Comparison of different adult stem cell types for treatment of myocardial ischemia. Circulation.

[80] Van der Bogt K.E., Schrepfer S., Yu J., Sheikh A.Y., Hoyt G., Govaert J.A., Velotta J.B., Contag C.H., Robbins R.C., Wu J.C. (2009). Comparison of transplantation of adipose tissue-and bone marrow-derived mesenchymal stem cells in the infarcted heart. Transplantation.

[81] Takaku Y., Murai K., Ukai T., Ito S., Kokubo M., Satoh M., Kobayashi E., Yamato M., Okano T., Takeuchi M. (2014). In vivo cell tracking by bioluminescence imaging after transplantation of bioengineered cell sheets to the knee joint. Biomaterials.

[82] Payne K.A., Lee H.H., Haleem A.M., Martins C., Yuan Z., Qiao C., Xiao X., Chu C.R. (2011). Single intra-articular injection of adeno-associated virus results in stable and controllable in vivo transgene expression in normal rat knees. Osteoarthr. Cartil..

[83] Mendelsohn A.R., Brent R. (1999). Protein interaction methods--toward an endgame. Science.

[84] Pfleger K.D., Eidne K.A. (2006). Illuminating insights into protein-protein interactions using bioluminescence resonance energy transfer (BRET). Nat. Methods.

